# Supplementary material for: Effect of the Pandemic Outbreak on ICU-Associated Infections and Antibiotic Prescription Trends in Non-COVID19 Acute Respiratory Failure Patients
Source: J Clin Med. 2022 Nov 29;11(23):7080. doi: 10.3390/jcm11237080 (PMC9739506; doi:10.3390/jcm11237080)

Supplemental Table S1. Multivariate Analysis on risk factors associated to total antimicrobial use.

	Unstandardized coefficient		Standardized coefficient	P value
	**B**	**Std Error**	**Beta**	
**Constant**	15.549	5.907		0.010
**SAPS II (value)**	-0.190	0.094	-0.203	*0.047*
**Heart Disease (yes)**	-1.528	2.770	-0.048	0.583
**Pneumopathy (yes)**	1.644	3.223	0.046	0.612
**DM (yes)**	-0.943	3.074	-0.026	0.760
**CKD (yes)**	5.558	3.986	0.139	0.167
**Year of admission (PP)**	8.868	2.851	0.280	*0.003*
**ICU stay (days)**	1.239	0.355	0.551	*0.001*
**IMV duration (days)**	-0.047	0.511	-0.015	0.927
**Acute respiratory failure after surgery (yes)**	-8.108	3.072	-0.256	*0.010*

Reference in parenthesis; PP=pre-Pandemic; SAPS II, Simplified Acute Physiology Score II; DM, diabetes mellitus; CKD, chronic kidney disease; Intra-Pandemic (2020); ICU, intensive care unit; IMV, invasive mechanical ventilation.

Supplemental Table S2: Microbial isolations in blood, respiratory tract and urinary tract samples, divided in families in the two years.

**PP: Pre-Pandemic**			**IP: Intra-Pandemic**
**FAMILIES**	**n. (%)**	**n. (%)**	**FAMILIES**
Staphylococcus spp.	23 (35.4)	18 (28.1)	*Candida spp. §*
Enterobacteriaceae	11 (16.9)	13 (20.3)	*Staphylococcus spp.*
Pseudomonas aeruginosa	10 (15.4)	9 (14.1)	*Others**
Others*	6 (9.2)	8 (12.5)	*Pseudomonas aeruginosa*
Acinetobacter baumannii	3 (4.6)	5 (7.8)	*Enterobacteriaceae*
Morganella morganii	3 (4.6)	5 (7.8)	*Stenotrophomonas maltophilia*
Streptococcus spp.	2 (3.1)	2 (3.1)	*Streptococcus spp.*
Candida spp.	2 (3.1)	2 (3.1)	*Enterococcus faecalis*
Stenotrophomonas maltophilia	2 (3.1)	1 (1.6)	*Klebsiella spp.*
Enterococcus faecalis	1 (1.5)	1 (1.6)	*Haemophilus influenzae*
Klebsiella spp.	1 (1.5)	0 (0)	*Acinetobacter baumannii*
Haemophilus influenzae	1 (1.5)	0 (0)	*Morganella morganii*
**Total number of isolated microorganisms**	65	64	Total number of isolated microorganisms

Legionella pneumophila, Proteus mirabilis, Corynebacterium spp., Serratia marcescens, Actinomyces, Lactobacillus casei, Citrobacter freundii, Alcaligenes xylosoxidans, Propionibacterium acnes. § Chi-square test with $p<0.05$ comparing the relative percentage in the two groups.

Figure S1: Relative percentage of isolated microorganisms' classes in the cumulative positive cultures, bloodstream cultures, respiratory tract cultures and urinary tract cultures in the two years of analysis. PP, pre-pandemic; IP, intra-pandemic.

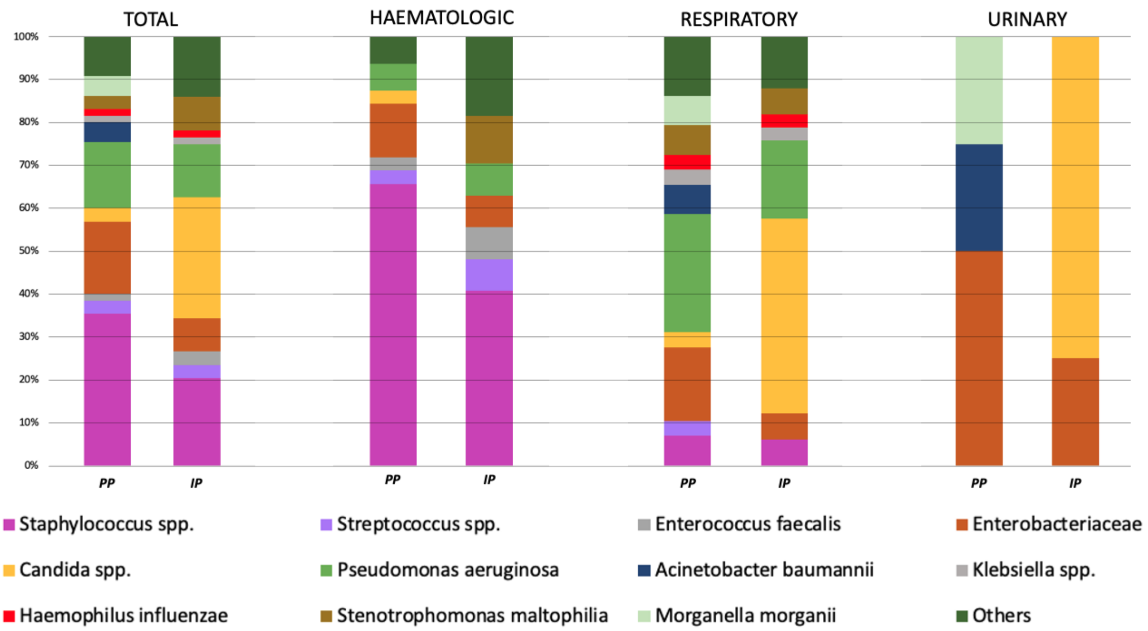

Supplement: Supplementary file 1 [file jcm-11-07080-s001.zip › jcm-2052929-supplementary.pdf]
